# Biomarkers and prediction of anthracyclic cardiotoxicity in breast cancer

**DOI:** 10.1590/1806-9282.2024S106

**Published:** 2024-06-07

**Authors:** Eduardo Nani Silva, Mario Luiz Ribeiro, Lilian Campos Caldeira, Antonio José Lagoeiro Jorge, Maria Luiza Garcia Rosa, Evandro Tinoco Mesquita, Humberto Villacorta, Wolney de Andrade Martins

**Affiliations:** 1 Universidade Federal Fluminense, Postgraduate Program in Cardiovascular Sciences – Niterói (RJ), Brazil.; 2 Rio Bonito Oncology Center – Rio de Janeiro (RJ), Brazil.; 3 Cardio-Oncology Postgraduate Program, Brazilian Society of Cardiology / National Institute of Cardiology / National Institute of Cancer – Rio de Janeiro (RJ), Brazil.

**Keywords:** Biomarkers, Cardiotoxicity, Chemotherapy, Anthracyclines, Breast cancer, Heart failure

## Abstract

**BACKGROUND:**

Chemotherapy with doxorubicin may lead to left ventricular dysfunction. There is a controversial recommendation that biomarkers can predict ventricular dysfunction, which is one of the most feared manifestations of anthracycline cardiotoxicity.

**OBJECTIVE:**

The aim of this study was to evaluate the behavior of biomarkers such as Troponin I, type B natriuretic peptide, creatine phosphokinase fraction MB, and myoglobin in predicting cardiotoxicity in a cohort of women with breast cancer undergoing chemotherapy with anthracycline.

**METHODS:**

This is an observational, prospective, longitudinal, unicentric study, which included 40 women with breast cancer, whose therapeutic proposal included treatment with doxorubicin. The protocol had a clinical follow-up of 12 months. Biomarkers such as Troponin I, type B natriuretic peptide, creatine phosphokinase fraction MB, and myoglobin were measured pre-chemotherapy and after the first, third, fourth, and sixth cycles of chemotherapy.

**RESULTS:**

There was a progressive increase in type B natriuretic peptide and myoglobin values in all chemotherapy cycles. Although creatine phosphokinase fraction MB showed a sustained increase, this increase was not statistically significant. Troponin, type B natriuretic peptide, myoglobin, and creatine phosphokinase fraction MB were the cardiotoxicity markers with the earliest changes, with a significant increase after the first chemotherapy session. However, they were not able to predict cardiotoxicity.

**CONCLUSION:**

Troponin I, type B natriuretic peptide, myoglobin, and creatine phosphokinase fraction MB are elevated during chemotherapy with doxorubicin, but they were not able to predict cardiotoxicity according to established clinical and echocardiographic criteria. The incidence of subclinical cardiotoxicity resulting from the administration of doxorubicin was 12.5%.

## INTRODUCTION

One of the most feared clinical presentations of cardiotoxicity resulting from cancer (CA) treatment is left ventricular dysfunction. In recent years, there has been a growing interest in the use of cardiac biomarkers (BMK) to guide the management of CA patients who will receive or are undergoing chemotherapy (CT) treatment against CA.

A biomarker is defined as a characteristic that is objectively measured and evaluated as an indicator of a normal, pathological, biological process or pharmacological response^1^. BMK, in addition to assisting in the diagnosis of cardiotoxicity in the pre-clinical phase, play a key role in guiding treatment strategies, including the initiation of "cardioprotection" during oncological treatment without compromising the therapeutic efficacy of CA^2^.

The literature relates cardiotoxicity to the occurrence of cardiomyopathy resulting from CT, radiotherapy (RT), and immunotherapy. In patients treated with anthracyclines (ANT), the measurement of left ventricular ejection fraction (LVEF) and global longitudinal strain (GLS) calculated by echocardiography have been the most commonly used tools in the assessment of cardiac function, although there is no consensus on whether the percentage decline in contractile function would represent a clinically relevant change that justifies prevention and intervention measures. In the last decade, the measurement of troponin I (TnI), type B natriuretic peptide (BNP), and its precursor, NT-pro-BNP, before and during CT treatment, proved to be a promising alternative in the early detection of cardiotoxicity as it is minimally invasive, easy to perform, without interobserver variability, and less expensive than imaging tests.

The European Society of Cardiology, in its recent Cardio-Oncology Guideline, recommended the measurement of BMK in the initial assessment of the risk of cardiotoxicity in patients with CA and cardiovascular disease or with previous ventricular dysfunction^3^.

This study aimed to evaluate the behavior of BMK such as TnI, BNP, creatine phosphokinase fraction MB (CK-MB), and myoglobin in predicting cardiotoxicity in a cohort of women with breast CA undergoing CT with ANT.

## METHODS

This is an observational, prospective, longitudinal, unicentric study, which included 40 women over 18 years old, with breast CA, whose therapeutic proposal included treatment with doxorubicin, from two high-complexity public oncology units, both in the State of Rio de Janeiro, Brazil. Patients with previous CT and/or RT, previous myocardial infarction, chronic obstructive pulmonary disease, chronic renal failure, and metastatic CA were excluded.

### Study population

**Figure 1:**
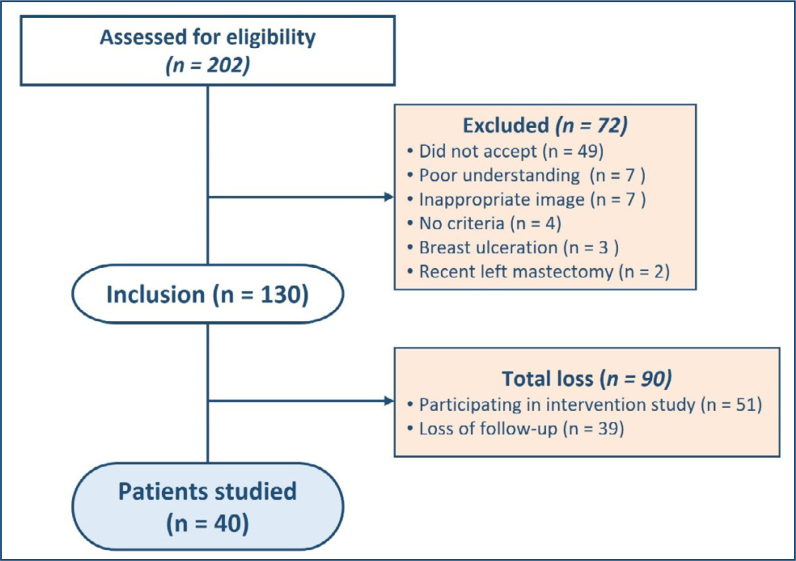
Study population.

A total of 202 patients were referred and evaluated, with 40 patients included in the final analysis. However, 72 patients were excluded from the study protocol in the initial assessment, of which 49 did not agree to participate. Furthermore, seven had an inadequate "echocardiographic window"; seven due to a low level of understanding of the study protocol; four because the inclusion criteria were not met; three due to ulceration of the left breast tumor, making echocardiographic examination unfeasible; and two due to recent surgery on the left breast, a reason that also makes the acquisition of echocardiographic images difficult. Among the 130 patients included, 90 subsequent exclusions occurred: 51 patients participating in another study underway at the same institution using carvedilol and 39 due to segment losses (Figure 1).

### Study protocol

**Figure 2:**
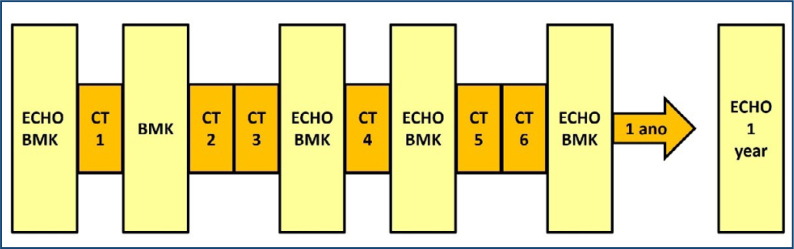
Study protocol. ECHO: echocardiography; BMK: biomarkers; CT: chemotherapy.

The study protocol had a clinical follow-up of 12 months. BMK such as TnI, BNP, CK-MB, and myoglobin were measured pre-CT and after the first, third, fourth, and sixth cycles of CT in a time interval between 24 h and 48 h after CT. BMK were not measured 12 months after starting CT, and 17 patients had only four sessions. Five patients did not undergo the 12-month evaluation: 3 deaths resulting from CA complications and 2 lost to follow-up (Figure 2).

### Biomarkers

To measure BMK, 5 mL of blood was collected from a peripheral vein and immediately added to the test panel on a point-of-care platform. The Alere Triage^®^ Cardiac Panel kit was used to measure CK-MB and TnI, and the Alere Triage^®^ BNP Test kit (Alere Inc., San Diego, California, USA) was used for BNP, using immunoassay methodology. The results were expressed in pg/mL for BNP and ng/mL for TnI, CK-MB, and myoglobin measurements.

### Ethical aspects

This study was conducted in accordance with the principles set out in the Declaration of Helsinki, revised in 2000. The study protocol was approved and monitored by the Research Ethics Committee, under the authorization of the National Research Committee, registration number 0084.0.258.000-11. All patients signed free, informed consent.

### Data analysis

Statistical analysis was performed using the SPSS version 21.0 software (Chicago, Illinois, USA). Continuous variables were expressed as means±standard deviation. Categorical variables were expressed as absolute numbers or percentages. For comparison between groups, the chi-square test was used to evaluate differences between proportions, the analysis of variance (ANOVA) between groups, and the Student's t-test to evaluate differences in means. In all comparisons, two-sided tests were considered with a significance level of 5%.

## RESULTS

### Demographic and clinical characteristics

Forty women with breast CA studied had a mean age of 55.9±10.8 years, with 42.5% white, 32.5% Afro-descendant, and 25.0% mixed race. The majority (77.5%) had a family income of up to two minimum wages. The patients presented high (77.5%), intermediate (12.5%), and low (10.0%) cardiovascular global risks. Among the cardiovascular risk factors studied, they had a sedentary lifestyle (85.0%), obesity and overweight (80.0%), hypertension (50.0%), dyslipidemia (37.5%), family history of arterial disease and premature coronary disease (37.5%), smoking (30.0%), and diabetes mellitus (20.0%).

The average systolic and diastolic blood pressure values found in the studied population were 143.9±19.3 mmHg and 87.6±13.2 mmHg, respectively. The average body mass index was 32.1±4.3 kg/m^2^.

The medications with cardiovascular and metabolic action most frequently used by the study patients were diuretics (32.5%), calcium channel blockers (22.5%), angiotensin-converting-enzyme inhibitors/angiotensin receptor blockers (ACEI/ARB) (17.5%), oral hypoglycemic agents (17.5%), and statins (12.5%).

### Cancer treatment data

Most patients (57.5%) used the FAC protocol (5-Fluoruracil+doxorubicin+cyclophosphamide), and 42.5% used the doxorubicin+cyclophosphamide+docetaxel protocol. The average dose of doxorubicin per session was 87.0±13.4 mg/m^2^, and the average cumulative dose used was 423.0±119.0 mg/m^2^. Cumulative doses ³400 mg/m^2^ were used in 24 patients (60%).

### Biomarker behavior

There was a progressive increase in BNP and myoglobin values in all CT cycles. Although CK-MB showed a sustained increase, this increase was not statistically significant. TnI had an increasing behavior during CT cycles, only reaching statistical significance in the last cycle of doxorubicin administration when compared to pre-CT values. For data analysis purposes, the mean biomarker values in CT4 and CT6 were grouped at the same time as they correspond to the last cycle of doxorubicin (Table 1).

**Table 1:** Comparison of mean biomarker values throughout treatment.

Variables	Pre-CT	Post-CT1	p-value	Post-CT3	p-value	Post-CT4/6	p-value
BNP (pg/mL)	33.7±22.8	52.6±41.2	0.003	68.6±66.7	0.010	72.9±56.6	**<0.0001**
Myoglobin (ng/mL)	47.9±26.9	60.2±22.7	0.017	62.4±28.1	0.007	64.5±24.3	**<0.008**
CK-MB (ng/mL)	0.77±0.59	0.85±0.62	0.511	0.92±0.56	0.083	0.95±0.63	0.067
TnI (ng/mL)	0.01±0.00	0.08±0.28	0.097	0.07±0.23	0.091	0.21±0.51	**0.030**

Statistically significant p-value are denoted in bold. CT: chemotherapy; CT1: first CT; CT3: third CT; CT4/6: fourth and sixth CT; BNP: type B natriuretic peptide; CK-MB: creatine phosphokinase MB fraction; TnI: troponin I.

### Biomarkers as predictors of cardiotoxicity

Echocardiogram examinations were performed in all patients pre-CT, post-CT3, post-CT4, post-CT6, and 12 months after the start of CT. In the present series, 5 patients (12.5%) developed asymptomatic systolic ventricular dysfunction with a drop in Simpson LVEF≥10% to values lower than 53%, 12 months after CT. GLS was also reduced by ≥10% in 2 patients, and TnI was positive during all CT sessions in only one patient, and none of the patients presented clinical heart failure syndrome.

The serum BMK such as troponin, BNP, myoglobin, and CK-MB were the cardiotoxicity markers with the earliest changes, with a significant increase after the first CT session. However, they were not able to predict cardiotoxicity in the studied population according to current clinical and echocardiographic criteria.

## DISCUSSION

There is still less evidence regarding the true predictive power of BMK for cardiotoxicity in different situations in cardio-oncology. Most recommendations are based on expert opinion. The largest clinical trial with BMK evaluated 703 patients treated with high doses of CT, including ANT, and followed them for an average of 20 months. TnI measurement was performed before the start of CT, after each cycle and 1 month after treatment, a methodology similar to that used in our study. This study demonstrated that patients who did not have TnI elevation during treatment and 1 month later had fewer outcomes related to cardiotoxicity (heart failure, asymptomatic LV dysfunction, or any other cardiac event) than those who had transient TnI elevations or who persisted with elevated TnI throughout clinical follow-up (1%, 37%, and 84%, respectively)^4^.

Type B natriuretic peptide has been used as a marker of ventricular dysfunction in CA-induced cardiotoxicity, with conflicting results^5^. Data from the Framingham study population demonstrate a progressive increase in the risk of cardiovascular death, heart failure (HF), stroke, and atrial fibrillation with a progressive increase in BNP levels, even within normal values^6^. Our study showed an ascending curve of BNP levels with statistical significance in all CT cycles (p<0.0001), despite not exceeding the cutoff value used to rule out HF in the emergency room, which is 100 pg/m. This behavior was also consistently reflected in the levels of myoglobin (p=0.008) and TnI (p=0.030). CKMB, despite having an ascending curve similar to the other BMK, did not reach statistical significance (p=0.067). These changes did not correlate with clinical cardiovascular outcome or with any echocardiographic parameter evaluating systolic or diastolic function throughout treatment. Only one of the five patients who presented asymptomatic ventricular dysfunction had a progressive increase in TnI throughout the treatment (pre-CT= 0.01 ng/mL; post-CT1=0.6 ng/mL; post-CT3=0.8 ng/mL; post-CT6=1.9 ng/mL).

The mean BNP value in the pre-CT assessment was 33.7±22.8 pg/mL, reaching significantly higher values immediately after the last cycle of doxorubicin (72.9±56.6 pg/mL, p<0.001). These values, despite being lower than 100 pg/mL, should be interpreted, considering current knowledge, as a high value for patients with stage A of HF. In a cross-sectional study that included 633 individuals from a primary healthcare program in the city of Niterói (RJ), Jorge et al.^7^ found 230 individuals (36.6%) in stage A of HF with an average BNP value of 19.7±21.2 pg/mL and a cutoff value of 42 pg/mL to exclude HF (negative predictive value of 99%). In our study, a higher mean BNP value may be related to the presence of a high global cardiovascular risk in 77.5% of patients and an intermediate global cardiovascular risk in 12.5% of patients.

BNP has been recommended in the most recent HF guidelines as a tool for detecting individuals predisposed to developing HF. The update of the first Brazilian Cardio-Oncology Guideline recommended the monitoring of BNP and TnI BMK for the purpose of early detection of cardiotoxicity at Class IIa level of evidence B^8^.

In our study, the mean BNP value in the pre-CT assessment of 33.7±22.8 pg/mL and its sustained increase during CT already signaled a population at risk for developing HF. Similar to what was observed in our study, Sawaya et al.^9^ demonstrated that high levels of NT-pro-BNP were not a predictor of reduced LVEF or the occurrence of symptomatic HF in a group of 81 women with HER2^+^ breast CA who used ANT followed by therapy adjuvant with taxanes and trastuzumab. Likewise, Fallah-Rad et al.^10^ in a prospective study using echocardiography, BMK [TnT, C-reactive protein (CRP), and NT-pro-BNP], and cardiac resonance in 42 patients undergoing adjuvant CT with trastuzumab, found no differences in the dosages of the three BMK studied between patients who developed cardiotoxicity (n=10) compared to those who did not have this complication.

Several other BMKs have been studied to detect cardiotoxicity. Putt et al.^11^ in a multicenter cohort study with 78 women with breast CA receiving doxorubicin and trastuzumab, evaluated the behavior of eight BMKs: TnI, NT-pro-BNT, Galectin 3, myeloperoxidase (MPO), factor placental growth factor (PIGF), growth differentiation factor-15 (GDF-15), soluble fms-like tyrosine kinase receptor 1 (sFlt-1), and high-sensitivity polymerase chain reaction (PCR), before the start of CT and every 3 months with a maximum follow-up of 15 months. All BMK, except NT-pro-BNP, increased in the third month after starting CT, persisting until the 15^th^ month for GDF-15, PIGF, and TnI. Elevations of MPO, PlGF, and GDF-15 correlated with the occurrence of cardiotoxicity, with MPO being the biomarker that presented a more robust correlation with the occurrence of cardiotoxicity in all assessments.

Several studies that included patients receiving CT with ANT followed by taxanes and trastuzumab did not demonstrate an association between BMKs CRP, Galectin-3, interleukin-1 receptor (ST-2), and GDF-15 and cardiotoxicity^12^.

In this study, the early and sustained rising behavior of BNP was the only parameter studied which was found to be correlated with the progression of the cumulative dose of doxorubicin administered.

## CONCLUSION

The cardiac BMK such as TnI, BNP, myoglobin, and CK-MB are elevated during CT with doxorubicin but they were not able to predict cardiotoxicity according to the established clinical and echocardiographic criteria.

The incidence of subclinical cardiotoxicity resulting from the administration of doxorubicin was 12.5%.
